# An Integrated Pipeline for the Genome-Wide Analysis of Transcription Factor Binding Sites from ChIP-Seq

**DOI:** 10.1371/journal.pone.0016432

**Published:** 2011-02-16

**Authors:** Eloi Mercier, Arnaud Droit, Leping Li, Gordon Robertson, Xuekui Zhang, Raphael Gottardo

**Affiliations:** 1 Computational Biology Unit, Institut de Recherche Clinique de Montreal, Montreal, Canada; 2 Department of Molecular Medecine, Faculty of Medicine, Endocrinology and Genomics, Centre de Recherche du CHUQ (CRCHUQ), Laval University, Quebec, Canada; 3 Biostatistics Branch, National Institute of Environmental Health Sciences, National Institutes of Health, Research Triangle Park, North Carolina, United States of America; 4 BC Cancer Agency, Genome Sciences Centre, Vancouver, Canada; 5 Department of Statistics, University of British Columbia, Vancouver, Canada; 6 Vaccine and Infections Disease Division, Fred Hutchinson Cancer Research Center, Seattle, Washington, United States of America; University of Georgia, United States of America

## Abstract

ChIP-Seq has become the standard method for genome-wide profiling DNA association of transcription factors. To simplify analyzing and interpreting ChIP-Seq data, which typically involves using multiple applications, we describe an integrated, open source, R-based analysis pipeline. The pipeline addresses data input, peak detection, sequence and motif analysis, visualization, and data export, and can readily be extended via other R and Bioconductor packages. Using a standard multicore computer, it can be used with datasets consisting of tens of thousands of enriched regions. We demonstrate its effectiveness on published human ChIP-Seq datasets for FOXA1, ER, CTCF and STAT1, where it detected co-occurring motifs that were consistent with the literature but not detected by other methods. Our pipeline provides the first complete set of Bioconductor tools for sequence and motif analysis of ChIP-Seq and ChIP-chip data.

## Introduction

Transcription factors (TFs) play critical roles in regulating gene expression. Determining transcription factor binding sites (TFBSs) is challenging because the DNA segments recognized by TFs are often short and dispersed in the genome, and the target loci of a TF vary between tissues, developmental stages and physiological conditions.

Genome-wide protein-DNA interactions are now typically profiled using ChIP-Seq, i.e. chromatin immunoprecipitation (ChIP) with massively parallel short-read sequencing [1]. A typical ChIP-Seq experiment generates millions of short (35–75 bp) directional DNA sequence reads that represent ends of ∼200 bp immunoprecipitated DNA fragments. The read sequences are mapped onto a reference genome. Then, for experiments with transcription factors, there are three central analysis issues: peak-calling, binding motif identification, and motif interpretation. Here, we report an R/Bioconductor-based pipeline that offers an efficient, integrated set of analysis tools for such experiments.

The aligned read data are first transformed into a form that reflects local densities of immunoprecipitated DNA fragments, and regions with high read densities, typically referred to as peaks, are identified by a peak-calling algorithm (Reviewed in [2], [3]). Here, we use an R package, based on PICS, which we developed for this pipeline. PICS (see methods) has been shown to perform well compared to the QuEST [4], MACS [5], CisGenome [6], and USeq [7].

Peak-calling returns a list of enriched genomic regions in which the protein of interest is expected to be directly or indirectly associated with DNA. Analysis then identifies potential DNA binding sites within these regions, and summarizes these sets of short sequences as motifs, typically as position weight matrices (PWMs) or families of PWMs [8], [9]. There are two main types of algorithms for *de novo* motif discovery: enumerative and probabilistic. Enumerative methods identify and rank all m-letter patterns in a set of sequences. Probabilistic methods use stochastic sequence models along with Expectation-Maximization (EM) or Gibbs sampling techniques to infer PWMs [10]–[13], and can be computationally impractical for large datasets. Established tools like Weeder [14], Gibbs sampler [15] or MEME [16] were developed to address relatively small sets of input sequences, and scale poorly to the much larger sets of enriched sequences that whole-genome ChIP-Seq data can return. Pipelines developed for ChIP-Seq analysis, e.g. CisGenome [6] and MICSA [17], are based on these algorithms or variants of them, and face similar constraints. Other tools like HMS [18] and ChiPMunk [19] were developed for motif discovery from ChIP-Seq data, and so are more scalable, but can identify only a single-motif at a time, and would need to be modified to discover motif combinations. Our pipeline uses GADEM [20], which is a good compromise between fully probabilistic and enumerative approaches, can process large sets of ChIP-Seq regions, handles both dimer and monomer motifs, automatically identifies multiple motifs, and automatically adjusts motif widths. We have ported GADEM to R, as a package called rGADEM. To address very large sets of enriched regions, we have extended the original C code to take advantage of multithreading, without requiring user configuration, via Grand Central Dispatch on OS X, and openMP (openmp.org), which supports shared-memory parallel programming on all architectures, including Unix and Windows. Compared to probabilistic approaches, this provides a simple, fast and efficient *de novo* framework.

Once *de novo* motifs have been identified, it is desirable to compare, annotate and assess these in order to retain motifs that are likely to be biologically relevant, while removing artifactual and background motifs. For this we have designed a new tool, MotIV (Motif Identification and Validation), which is based on STAMP [21]. Like STAMP, MotIV provides queries to the JASPAR database [22], and users can flexibly input other sets of reference PWMs (e.g. TRANSFAC [23], UniProbe [24], DBTBS [25], or RegulonDB [26]). As outlined below, MotIV provides visualization and postprocessing options that are unavailable in STAMP, TOMTOM [27] and MACO [28]. It provides summary statistics on motif occurrences, reports joint motif occurrences and plots distance and pairwise-distance distributions. It can also refine motifs and motif occurrences based on a set of filters provided by the user.

Because gene regulation typically involves combinatorial action of multiple TFs, functional binding sites tend to occur as groups that are often referred to as cis-regulatory modules (CRMs) [29]. Identifying CRMs can improve the accuracy of predicting functional binding sites. However, results from computational methods for determining CRMs (e.g. Cluster-Buster [30] and CisModule [31]) are rarely reported for ChIP-Seq data, because they are too computationally intensive or return long lists of candidate modules that are challenging to assess. MotIV offers an alternative way to identify biologically relevant combinations of motifs.

Below, we describe the pipeline in more detail. Its core consists of three Bioconductor packages: PICS calls enriched regions; rGADEM identifies *de novo* motifs; and MotIV visualizes and annotates motifs, and identifies motif combinations that have nonrandom spatial relationships. This is the first complete Bioconductor pipeline for analyzing transcription factor ChIP-Seq data. The pipeline is computationally efficient, supporting processing datasets that consist of tens of thousands of peaks. We illustrate the pipeline by analyzing published Illumina datasets for genome-wide binding in human of FOXA1, ER, STAT1, and CTCF. We compare the performance of our approach to previously described methods for motif and module discovery, and show that the pipeline supports detecting biologically relevant motif modules that are not easily discovered by other methods.

## Results

We applied the pipeline to the four ChIP-Seq datasets mentioned above and described in the Methods section. We first used PICS to select the top 15000 enriched regions for the CTCF, STAT1 and the FOXA1 data. For the STAT1 and FOXA1 data, this corresponded roughly to a 5–10% FDR. For the ER data, PICS detected 8000 enriched regions at a similar FDR level (Figures S1, S2, S3). For CTCF, because we had no control data, we used the top 15,000 regions for consistency with STAT1 and FOXA1. In each case, we used PICS to export the top-ranked 400-bp wide enriched regions around predicted binding sites (peak centers). In R, this creates a *RangedData* object, containing the chromosome, start and end positions of each sequence, which can be input directly into rGADEM. We post-processed the resulting rGADEM object using MotIV.

### Identification of primary motifs

rGADEM respectively identified 68, 23, 25 and 78 motifs in the CTCF, STAT1, FOXA1 and ER datasets. To interpret the detected motifs, we used MotIV to compare the identified PWMs to JASPAR PWMs [22]. For each input motif, MotIV returns a user-defined number of best-matching PWMs from the user-specified reference database. The output consists of the name and sequence logo of the highly-ranked database hits, along with the pairwise alignments (in consensus sequence format) and the alignment E-values (see Figures S4, S5, S6, S7, S20).

**Figure 1:**
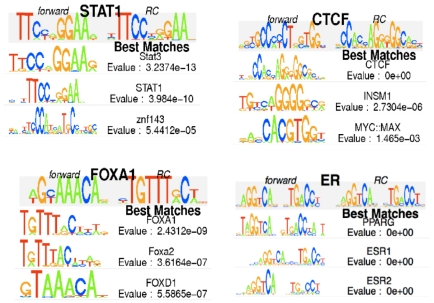
Primary motifs identified by rGADEM and visualized with MotIV. The motif matches and associated similarity E-values are based on the JASPAR database included in MotIV.

**Figure 2:**
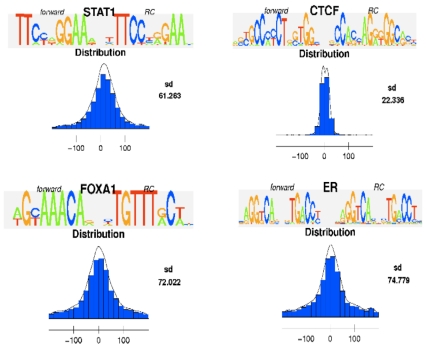
Distance distribution between the rGADEM motif occurrences and the PICS predictions for the STAT1, CTCF, FOXA1 and CTCT motifs identified from datasets.

When displaying PWM matches, the user can choose to set filters that retain only certain motifs, e.g. all matches with an E-value less than 10^−4^, or all matches containing the name ‘STAT’. Here, we retained only the ‘expected’ motif for each data set (Figure 1) by filtering on the names STAT1, CTCF, FOXA1 and ESR1 in the JASPAR database and applying an E-value cutoff of 10^−4^. Figures S4, S5, S6, S7 show that rGADEM can sometime identify variants of the same motif (e.g. FOXA1). A user may chose to combine the motif occurrences of these variants and treat them as occurrences of the same motif. This can easily be done via MotIV's *combine* method, which regroups multiple motifs based on a set of filters. Using this approach, we combined all variants of primary motifs, as follows: FOXA1 = {m5,m10,m25}, ER = {m4,m22,m33,m48}, STAT1 = {m1} and CTCF = {m1}. Note that such combining is ‘virtual’, in that the PWMs of the selected motifs are not actually combined nor modified, but are simply assigned the same label. We find the combining process particularly useful for plotting distributions and exporting motif occurrences, and the interactive R environment readily supports iteratively exploring such operations. As a secondary check, we used the distance distribution plots provided by MotIV. Given the specificity of ChIP-Seq and the accuracy of PICS, a *de novo* motif that reflects a DNA-binding interaction should be located close to a PICS site prediction. Using both the output of rGADEM and the *RangedData* object returned by PICS (i.e. the input of rGADEM) MotIV can plot the frequency distributions of the distance between motif occurrences and peak centers. Note that such distance distribution plots do not depend on database matches, and so can be used with novel motifs and motif variants. Figures S8, S9, S10, S11 shows that the selected motifs are concentrated around peak centers, as expected. Our combined primary motifs resulted in a total of 10059, 7105, 8711 and 3947 binding site occurrences for CTCF, STAT1, FOXA1 and ER respectively. Figure 2 shows the distribution for the combined primary motifs. Overall, the spatial error between PICS binding site predictions and actual motif occurrences is relatively small.

### Identification of secondary motifs

**Figure 3:**
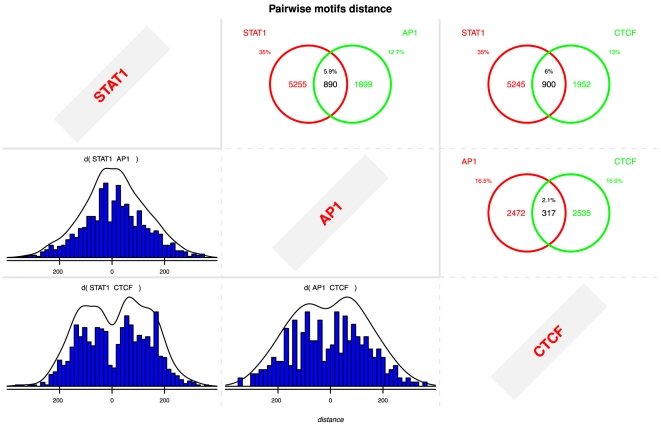
Pairwise distance distributions between the STAT1, AP-1 and CTCF motifs identified by rGADEM from the STAT1 data.

Once expected motifs have been identified, we now look for other motifs that may be biologically relevant. Because we may not know which secondary motifs to expect, further computational assessment may be required to discriminate artifactual motifs. A simple but elegant approach involves using distributions of distances between rGADEM motif occurrences and PICS predicted binding sites. If the identified motif corresponds to a protein that has a short-range interaction with the immunoprecipitated protein, we would expect the motif site to be close to the PICS site prediction. A quick look at the distribution plots, sequence logos and E-values reveals three interesting motifs for the STAT1 data: STAT1, AP-1 and CTCF (Figure 3 and Figures S7, S8, S9, S10, S11). A similar approach suggested ER, FOXA1 and AP-1 motifs for the ER data, FOXA1 and AP-1 for the FOXA1 data, and CTCF and Myf for the CTCF data. As noted above, we identified 68 and 75 motifs for CTCF and ER respectively. MotIV let us quickly filter and visualize these (Figures S4, S5, S6, S7, S8, S9, S10, S11), and suggested that many of these were either variants of the same motif or artifactual motifs due to sequence repeats.

MotIV also provides a way to characterize how frequently two motifs occur on the same input sequence, as well as distance distributions between occurrences of any two motifs. Figure 3 and Figures S12, S13, S14 show that there were fewer secondary motifs than primary, and that relatively large fractions of a secondary motif's sites can co-occur with a primary motif. The distance distributions show that most distances between a primary motif and its secondary ones are relatively short (∼50–100 bps), suggesting that the DNA-associated proteins may interact.

### Functional annotation of motifs and modules

To complement our analysis, we can combine our results with Gene Ontology (GO) annotations [32], using R's ChIPpeakAnno, to provide general insights into the functions of proteins targeted by ChIP-Seq experiments. For primary motifs, we identified several over-represented terms for associated genes, as determined by the nearest transcriptional start site (TSS) (Tables S1, S2, S3, S4). In general, the categories for the primary motifs listed in the tables were consistent with the known biological role of ER/FOXA1/AP1 (see Supplementary Material S1). Applying the same analysis looking at genes that were close to motif pairs formed by the primary motif and one secondary motif (Tables S1, S2, S3, S4) returned terms that, in some cases, were not returned when working with primary motifs only, which suggested that motif pairs may be functionally more discriminatory.

### Biological significance of modules

Given that PICS, rGADEM and MotIV support efficiently identifying candidate factor-cofactor relationships in ChIP-Seq data, we assessed whether the literature suggested that the relationships identified were biologically meaningful. FOXA1, which is regulated in response to estrogen treatment, has been shown to be crucial for ER to bind to chromatin and activate target gene transcription [33], [34]. This supports the FOXA1 motif detected by rGADEM in ER-enriched regions, and supports an interaction between the two proteins.

Fos and Jun family proteins usually function as dimeric transcription factor that bind to AP-1 regulatory elements [TGA(C/G)TCA] [35], [36]. The AP-1 complex has been shown to be over-expressed in ER positive cells (e.g. MCF7) and can interact directly with the ER transcription factor [37], [38]. This supports the AP-1 motif identified by rGADEM in the ER enriched regions, and the AP-1 motif that we identified in FOXA1-enriched regions, which may reflect interactions, possibly indirect, between the AP-1 and FOXA1 proteins via ER.

Given that we identified the FOXA1 motif in the ER-enriched regions, we expected to identify the ER motif in the FOXA1-enriched regions. We noted that a previous attempt to discover the ER motif in this dataset had been unsuccessful [5]. A seeded analysis with rGADEM (see Methods), using the ESR1 motif from JASPAR, identified an ER motif (Figure S15) with only 723 sites. These results suggest that ER requires FOXA1, but that the converse is not true, which is consistent with the above literature. Additionally, only 7% of the ER sites identified in the ER-enriched regions overlapped with a FOXA1-enriched region. For this calculation we used MotIV to export the ER sites as a *RangedData* object and used the *countOverlaps* function of the IRanges package to count the number of such sites that overlapped a FOXA1 enriched regions.

We examined the predicted interaction between STAT1 and AP-1 (Table 1 and Figure 3). Cytokine stimulation induces members of the STAT transcription factor family, Stat1 and Stat3, to ‘dock’ onto receptor phosphotyrosines, enabling their own tyrosine phosphorylation [39]–[41]. Subsequently, STAT proteins translocate to the nucleus and bind to conserved genomic regulatory sequences to rapidly activate gene transcription [42], [43]. The cytokines also activate components of other intracellular signaling pathways, including Ras, mitogen-activated protein kinase (MAPK), and the Fos-Jun (AP-1) transcription factors [44]–[46], and activate direct interaction between STAT1 and AP-1 [47]. This supports our AP-1 motif detected by rGADEM in the STAT1 enriched regions.

**Table 1:** Motifs identified by all compared methods.

	CTCF	ER	FOXA1	STAT1
rGADEM	CTCF (0)	ER (0)	FOXA1 (2e-12)	STAT1 (3e-13)
	Myf (4e-8)	FOXA1 (5e-12)	AP1 (6e-10)	CTCF (0)
		ETS-like (1e-8)		ETS-like (9e-7)
		AP1 (3e-7)		AP1 (6e-10)
cisFinder	CTCF (0)	ER (0)	FOXA1 (4e-13)	STAT1 (2e-10)
		ETS-like (9e-8)		AP1 (9e-8)
		AP1 (8e-3)		
Flexmodule	CTCF (0)	ER (0)	FOXA1 (3e-11)	STAT1 (4e-11)
		FOXA1 (1e-13)	AP1 (4e-8)	SRF (1e-8)
				AP1 (3e-8)
Weeder	CTCF (2e-11)	ER (1e-14)	FOXA1 (1e-12)	STAT1 (2e-11)
				AP1 (1e-10)
				ETS-like (2e-8)
MEME	CTCF (0)	ER (0)	FOXA1 (2e-15)	STAT1 (5e-9)
		AP1 (3e-4)		ETS-like (1e-5)
				AP1 (4e-4)

Motifs identified by all compared methods in the selected PICS enriched regions. The number given between parenthesis is the E-value match to the corresponding JASPAR motif.

We analyzed the predicted interaction between CTCF and Myf (Table 1). Wilson and *al.* [48] suggested that CTCF binding is required for MyoD-induced IGF-2 gene activity in muscle. Moreover, Myf and CTCF can co-localize in the same cellular fraction during cellular process [49]. In this case the literature is not as supportive but does suggest a potential co-operation between CTCF and Myf.

Finally, we found no strong evidence in the literature for an interaction between CTCF and STAT1. Given this, we again used MotIV to export the CTCF sites as a *RangedData* object and used the *countOverlaps* function of the IRanges package determine that 28% of such sites overlapped a CTCF enriched region, even though the two experiments used different cellular systems. A similar analysis showed that 31% of the FOXA1 sites identified in the ER-enriched regions overlapped with a FOXA1-enriched region. Note that such intersections of genomic intervals can easily be carried out using the *RangedData* class and methods provided by the IRanges package, illustrating how Bioconductor and R can be used to extend our pipeline.

### Comparison with other methods

In order to assess the performance of our pipeline, we compared other motif/CRM identification tools on the above four datasets, using PICS for peak calling and MotIV for validation.

CisFinder and Cluster-Buster took less than a minute on 15000 sequences, while Weeder and FlexModule took several days. Using 8-core multithreading, rGADEM completed these runs in a few hours. MEME's computational requirements allowed us to process only the top 5000 sequences for all datasets, even when using the parallel version running on 24 CPUs. HMS and ChIPMunk return a single motif from a run, and so are less directly applicable for work involving combinations of motifs. As well, while they are scalable, they are slower than rGADEM; for motif discovery on 15000 400-bp sequences, HMS (100 iterations) and ChIPMunk took approximately 24 h on a 16×2.4 Ghz server.

The number of motifs identified varied greatly between the *de novo* motif analysis tool (Table 1). As expected, each method returned the primary or expected motif from each dataset, and the methods compared agreed relatively well for these motifs (Figures S16, S17, S18, S19). The *de novo* tools differed in the secondary motifs and modules identified (Table 1). Weeder and CisFinder systematically returned the lowest number of motifs, while rGADEM and MEME tended to identify larger numbers of secondary motifs. rGADEM identified the most secondary motifs that could all be supported from the literature.

Cluster-Buster identified, in average, 1587 clusters containing 12 motifs for ER, 4558 clusters containing 15 motifs for CTCF, 1484 clusters containing 12 motifs for STAT1 and finally 1501 clusters containing 16 motifs for FOXA1. Such large numbers of motifs and clusters are difficult to interpret, and complicates comparison with other methods. While Cluster-Buster identified the same motif combinations as our pipeline in some of its clusters, these were mixed with tens of other motifs in thousands of clusters, validation of which would clearly be difficult. Additionally, cisFinder and Cluster-Buster used the input PWMs to scan for motif occurrences, and so assume that these motifs are sufficiently representative. In contrast, MotIV uses PWMs only for ‘labeling’ motifs.

## Discussion

We have developed a pipeline for analyzing ChIP-Seq data for transcription factors, the core of which consists of three complementary R packages: PICS, rGADEM and MotIV. Using four published human datasets, we showed that the pipeline compares favorably to other *de novo* motif tools and CRM clustering tools. For example, it identified co-occurring pairs of motifs that were consistent with the literature and were not detected by other methods.

Other integrated pipelines for ChIP-Seq data are available, for example, MICSA [17], CEAS [50], and Sole-Search [51]. Issues that should be considered in assessing such systems are reviewed by [52]. MICSA [17] was largely designed to improve ChIP-Seq data analysis by prioritizing enriched sequences that contained a motif logo for the expected motif. In MICSA, the authors use MEME on the top few hundred sequences to detect *de novo* motifs, and then scan the remaining sequences with the identified logos. While this can improve the speed of motif discovery, its biased subsampling of input sequences may compromise detecting secondary motifs. CEAS [50] and SoleSearch [51] are largely annotation systems that offer less functionality and are less flexible than our pipeline. We briefly tried to compare CEAS to our pipeline, but, as with Cluster-Buster, found this difficult because of the lack of control over the output.

The R pipeline described here offers functionality that is not available in CEAS, cisGenome, MICSA and Sole-Search, e.g. distance distribution plots, pairwise distance plots and motif filtering. Filtering is efficient in removing artifactual and background motifs based on combinations of E-values and distance distributions. For this reason, for our pipeline it is unnecessary to mask sequence repeats, which is recommended for CEAS and MICSA. As such masking could remove informative motifs, an unmasked approach may be preferable. Other approaches (e.g. cisGenome [6], [53]) use relative enrichment computed using control regions to discriminate relevant motifs from irrelevant ones. rGADEM reports a fold enrichment for each motif, and the pipeline complements this metric with information on distance distributions and pairwise separation distributions.

Although the methodology behind PICS, and an earlier command line version of GADEM have been published and demonstrated elsewhere, MotIV was developed for the pipeline, and the PICS and rGADEM R packages are new and implement improved versions of the respective algorithms. All novel computational aspects of rGADEM are described in Supplementary Material S1. While for the work reported here we focused on ChIP-Seq experiments, rGADEM and MotIV can also be used with ChIP-chip data. The pipeline provides our rMAT package [54], which is well integrated with rGADEM and MotIV, in that rMAT can export enriched regions as *RangedData* objects that can directly be input into rGADEM. The pipeline's modularity makes it straightforward to replace PICS with an alternative peak caller, and rGADEM with an alternative motif finder. Because our implementation is open-source, anyone with a basic knowledge of R can make such modifications.

**Figure 4:**
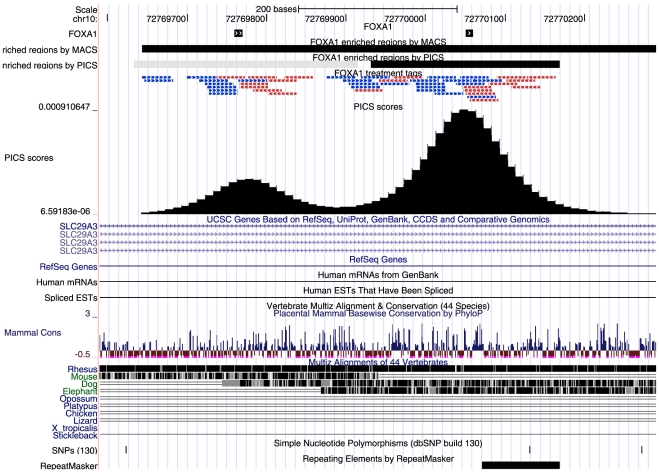
PICS peak calling. The example shows a FOXA1-enriched region in which PICS discriminates two closely adjacent binding events, each of which contains a rGADEM *de novo* FOXA-like motif (black squares); these are separated by less than 300 bps. In contrast, MACS outputs a single enriched regions. For clarity, the aligned reads (blue/red bars) and the combined forward/reverse PICS density profiles are also shown.

Finally, we emphasize that our pipeline can leverage other Bioconductor packages so that a user can develop, repeat and share advanced analyses. We have described some of these packages, but there are many more libraries that could be used with our pipeline. For example, Figure 4 makes use of the rtracklayer package [55] to interact with the UCSC genome browser. Other packages that can be used include: SeqLogo for visualization of PWM, GenomeGraphs [56] for further graphics functionality, BiomaRt for retrieving annotations, IRanges and GenomicRanges for interval manipulations, Biostrings for sequence manipulations, etc. Many other relevant packages are listed on the Bioconductor website. We anticipate that the characteristics of the R environment, including its extensibility, will help to make the pipeline useful for a wide range of ChIP-Seq datasets.

## Materials and Methods

**Figure 5:**
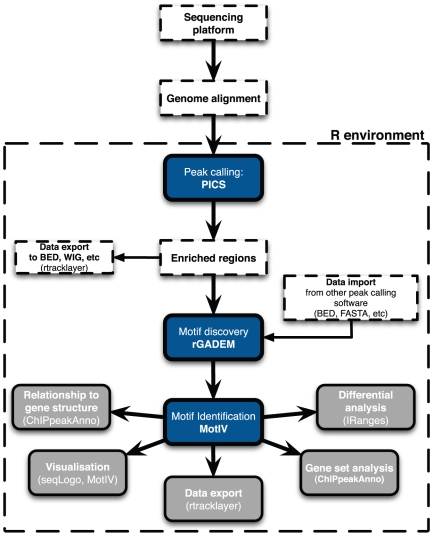
The ChIP-Seq processing pipeline. Short sequence reads are first mapped onto a reference genome, and the mapping results are loaded into R. The pipeline core consists of the three dark blue rectangles. Enriched regions are identified by PICS and passed to rGADEM for *de novo* motif discovery, and motifs and motif occurrences are passed to MotIV for postprocessing.

The analysis pipeline consists of three main steps (see Figure 5): peak calling, motif discovery, and motif postprocessing and validation. These steps are handled by three R packages: PICS, rGADEM and MotIV, which have been designed to work together and interact with other Bioconductor packages.

### Peak calling: PICS

**Figure 6:**
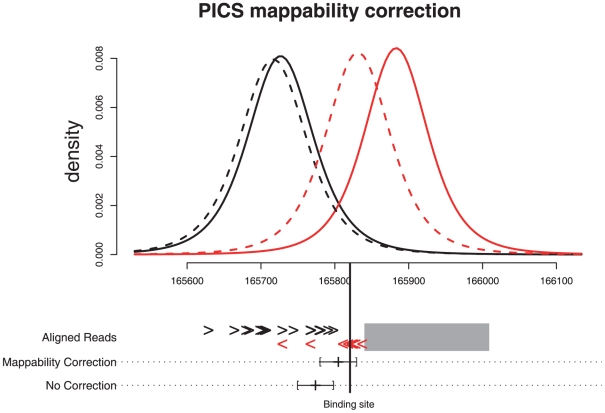
PICS read mappability correction in a FOXA1 binding region with missing reads due to genome repetitiveness. A non-mappable region (i.e. a region into which short reads cannot be uniquely mapped) is shown as a grey rectangle. Forward and reverse aligned reads are respectively shown as black and red arrowheads. Forward and reverse PICS read density profiles are respectively shown in black and red, with solid/dashed lines representing t distributions with/without the mappability correction. The rGADEM -estimated FOXA1 binding site is shown by a vertical black line. When PICS corrects for read mappability, the *de novo* motif is within the confidence interval of the site location that it predicts, but it is outside of the interval when the correction is not used. The spatial error, i.e. the distance between binding site location and the PICS prediction, is 15 bps with the correction and 47 bps without the correction.

The first step consists of identifying, from the aligned ChIP-Seq reads, regions that represent protein-DNA association. For this step, we rely on our method, PICS [57]. PICS is based on a Bayesian hierarchical truncated *t*-mixture model, and integrates four important components. It jointly models local concentrations of directional reads. It uses mixture models to distinguish closely-spaced adjacent binding events. It incorporates prior information for the length distribution of immunoprecipitated DNA to help resolve closely adjacent binding events (see Fig. 4), and identifies enriched regions that have atypical fragment lengths. Finally, it uses pre-calculated whole-genome read “mappability” profiles to adjust local read densities that are missing due to genome repetitiveness (see Fig. 6 and “Availability”, below). When a negative control sample is available (e.g. input DNA), PICS returns an enrichment score that is relative to the control sample for each binding event. Given a control sample, PICS can also estimate a false discovery rate (FDR) as a function of the enrichment score, which can be used to select a threshold score for segmenting (calling) enriched regions. Because PICS is based on a formal statistical model that requires an EM algorithm for estimating the unknown parameters, we have designed the R package PICS to be computationally efficient enough to process large sets of ChIP-Seq reads. The core of the algorithm is coded in C, and a user can easily take advantage of parallel processing via R's snowfall [58] and multicore packages.

Figure 6 illustrates the read mappability correction in a genomic region from the FOXA1 data. With the correction, the estimated PICS binding site was within the PICS 95% approximate confidence interval for the FOXA1 binding site location identified by rGADEM; when no correction was done, the de novo motif was outside of this interval. Figure 4 also shows that PICS can discriminate closely adjacent binding events. Two binding sites are separated into two disjoint enriched regions by PICS, whereas MACS [5] combined these two sites into a single region. Such features make PICS particularly attractive for subsequent motif-based analyses.

### *de novo* motif discovery: rGADEM

From the list of enriched regions returned by PICS, the next step involves discovering over-represented DNA motifs. Probability model-based *de novo* motif finding algorithms like MEME can be sensitive [59], [60], but may be too slow when thousands to tens of thousands of enriched regions need to be analyzed.

We have developed an open-source R package rGADEM, based on the GADEM software [20]. GADEM is an efficient and scalable *de novo* motif discovery tool that combines spaced dyads and an expectation-maximization (EM) algorithm. A genetic algorithm (GA) guides the formation of a “population” of spaced dyads. Each spaced dyad is converted into a letter probability matrix, which is optimized by an EM algorithm. The optimized PWM is then used to scan for binding sites in the data. A subsequence of the length of the PWM is declared a binding site when the *p*-value of its PWM score is less than or equal to a preset threshold value. The logarithm of the E-value [61]–[63] is used as the fitness score for the spaced dyad from which the motif is derived. The resulting unique motifs with fitness values less than or equal to a pre-specified cutoff are reported, and corresponding binding sites in the original sequences are masked. This procedure is repeated until no further motifs can be found that satisfy the run parameters.

rGADEM is an R package containing an extended version of the original GADEM C code. For ChIP-Seq data, a key improvement is that, on multicore computers, it can take advantage of multithreading via Grand Central Dispatch on Mac OS X 10.6 and above, and openMP on other Unix platforms, to sharply reduce run times.

A second important extension, shared by both R and the current command line versions, is an optional ‘seeded’ analysis run mode. In this mode, rGADEM does not generate the starting PWMs through spaced dyads, but instead initializes the optimization with a user-specified PWM. This PWM guides motif discovery, but is used only for initialization and not during the EM-based PWM updating. A seeded analysis has two important advantages. It is approximately ten times faster than an standard run. Further, the prior knowledge helps address both signal-to-noise issues [64] and problematic (e.g. short) motifs. In our experience, seeded runs are also useful for ChIP-chip data, where the signal is less clear and expected motifs can be more difficult to recover.

rGADEM can also prioritize sequences with large ChIP enrichments and includes novel prior distributions that prioritize for motif occurrences that are nearer to sequence (peak) centers. Such prior settings can potentially improve the detection of primary motifs at the cost of missing secondary motifs that can be present at low enrichment and/or further away for the center. For these reasons, we prefer to use the default uniform prior and use our post processing tools to detect biologically relevant motif combinations.

All novel computational aspects of rGADEM are described in Supplementary Material S1. Because the C code has been wrapped in R, the overall interface is accessible and the package contains functions to ease manipulation and visualization of the input and output.

### Post-processing and motif interpretation: MotIV

To identify a subset of potentially biologically relevant *de novo* motifs, we have developed a simple, efficient post-processing tool, MotIV. Based on STAMP [21], it compares and annotates motifs, and supports identifying candidate motif modules. MotIV accepts as input an R object returned by rGADEM, a PWM output file from the command-line version of GADEM, or a PWM in TRANSFAC format [23]. MotIV can be used to compare a list of input motifs against a reference motif database. It contains the JASPAR 2010 database, with pre-computed stimulated profiles that are used to determine the likelihood or E-value of a motif similarity score (see [21] for details). User-supplied PWM databases and can easily be used, and scores computed. Because MotIV uses the STAMP source code, it provides a range of options for alignment calculations (see the documentation for the R package and/or STAMP).

MotIV also provides several new visualization functionalities for sequence logos, motif occurrence distributions and pairwise distance distributions, which are available in grid layouts (Figures S4, S5, S6, S7, S8, S9, S10, S11, S12, S13, S14, S15). The first type of plot displays the alignment as logos with motif similarity E-values for the top 5 matches (this number can be changed). Because sequence repeats in artifactual enriched regions (e.g. regions that have high fractional overlaps with simple tandem repeats [65]) can lead to the detection of motifs with good E-value matches, MotIV provides several options for identifying and filtering such artifactual motifs. For example, MotIV allows one to plot the distribution of the motif occurrences within our enriched regions. A biologically relevant motif should have a distribution that is peaked around the center of the region; conversely, the spatial distribution for a less relevant motif will typically be flatter.

Finally, in order to identify co-occurring combinations of motifs, MotIV can display motif pairwise distance distributions. In such a plot, one can quickly quantify both co-occurring motif pairs and assess the distribution of the inter-motif distances. To our knowledge, no other method provides such functionality. Once interesting motifs have been identified, motifs and motif occurrences can easily be filtered and exported for further analysis. Note that for motif occurrence and pairwise distributions, the use of a database is not required, and novel motifs can be discovered based on their spatial distributions alone.

### Software availability and architecture

In the three packages, the source code is written in C for speed, and wrapped in R code for accessibility. All packages use object-oriented programming with classes and methods, which supports usability as well as integration with other R/Bioconductor packages [66], making it straightforward for a user to construct advanced analyses. For example, PICS and MotIV support exporting enriched regions and MotIV occurrences as *RangedData* objects which can directly be used by other packages such as ChIPpeakAnno [67], BSgenome and rtracklayer [55].

PICS, rGADEM and MotIV are available from the Bioconductor web site at http://bioconductor.org. They run on Linux, OS X ad MS-Windows. The packages are distributed under the terms of the Artistic License 2.0. Each contains a detailed manual and vignette with examples. Frequently asked questions, additional tutorials, and further installation instructions can be found at http://wiki.rglab.org. In addition, we offer pre-generated mappability profiles for common genomes and read lengths, as well as a “proMap” pipeline that can be installed locally for generating such profiles (http://wiki.rglab.org/index.php?title=Public:Mappability_Profile). The profiles are based on aligning read-length segments of a reference genome back to that reference genome, using the same aligner (BWA, [68]) and parameters that we use for ChIP-seq data.

### Data sets

To demonstrate the power and resolution of analyses supported by our pipeline we used four recently published ChIP-Seq data for human transcription factors: CTCF (CCCTC-binding factor) in CD4+ T cells [5], STAT1 in interferon stimulated (IFN-gamma) HeLa S3 cells [69], and FOXA1 [5] and Estogen Receptor in the MCF-7 breast cancer cell line [70]. The CTCF data contains 2.95M reads, the STAT1 data contains 26.7M treatment reads and 23.4M input control reads, the FOXA1 data consists of 3.9M treatment reads and 5.2M input control reads, and finally the ER data contains 3.6M treatment reads and 5.2M input control reads.

### Comparison to other methods

Because we have already shown that PICS compares favourably to other peak finders [57], we considered only steps 2 and 3 for comparing to other *de novo* motif tools. Because STAMP is widely used for motif postprocessing and MotIV extends STAMP, we used MotIV for step 3. Essentially, then, we were largely comparing rGADEM with other *de novo* discovery tools, for which we used MEME, cisFinder [71], FlexModule [6] and Weeder [72], which are widely used and perform well. For module discovery we compared our pipeline to Cluster-Buster [30]. Each application was used with its default parameters, according to the instructions given in the manuals. All computations were performed on a Mac Pro with dual 3.2 Ghz Quad-Core CPU processors and 16 GB RAM.

## Supporting Information

Figure S1 **Estimated FDR as a function of the enrichment score for the ER data.** The number of enriched regions for the corresponding score is given at the top.
(EPS)

Figure S2 **Estimated FDR as a function of the enrichment score for the FOXA1 data**. The number of enriched regions for the corresponding score is given at the top.
(EPS)

Figure S3 **Estimated FDR as a function of the enrichment score for the STAT1 data.** The number of enriched regions for the corresponding score is given at the top.
(EPS)

Figure S4 **Motifs identified by rGADEM and visualized with MotIV from the CTCF data**. The motif matches and associated E-values are based on the JASPAR database included in MotIV. For clarity only motifs with E-value less than 10^−4^ are retained.
(TIFF)

Figure S5 **Motifs identified by rGADEM and visualized with MotIV from the ER data.** The motif matches and associated E-values are based on the JASPAR database included in MotIV. For clarity only motifs with E-value less than 10^−4^ are retained.
(TIFF)

Figure S6 **Motifs identified by rGADEM and visualized with MotIV from the FOXA1 data.** The motif matches and associated E-values are based on the JASPAR database included in MotIV. For clarity only motifs with E-value less than 10^−4^ are retained.
(EPS)

Figure S7 **Motifs identified by rGADEM and visualized with MotIV from the STAT1 data.** The motif matches and associated E-values are based on the JASPAR database included in MotIV. For clarity only motifs with E-value less than 10^−4^ are retained.
(EPS)

Figure S8 **Distance distribution between the rGADEM motif occurrences and the PICS predictions for all motifs identified in the CTCF data.** For clarity only motifs with E-value less than 10^−4^ are retained.
(EPS)

Figure S9 **Distance distribution between the rGADEM motif occurrences and the PICS predictions for all motifs identified in the ER data.** For clarity only motifs with E-value less than 10^−4^ are retained.
(EPS)

Figure S10 **Distance distribution between the rGADEM motif occurrences and the PICS predictions for all motifs identified in the FOXA1 data.** For clarity only motifs with E-value less than 10^−4^ are retained.
(EPS)

Figure S11 **Distance distribution between the rGADEM motif occurrences and the PICS predictions for all motifs identified in the STAT1 data.** For clarity only motifs with E-value less than 10^−4^ are retained.
(EPS)

Figure S12 **Pairwise distance distributions between the CTCF, Myf motifs identified from the CTCF data.**
(EPS)

Figure S13 **Pairwise distance distributions between the ER, FOXA1 and AP-1 motifs identified from the ER data.**
(EPS)

Figure S14 **Pairwise distance distributions between the FOXA1 and AP-1 motifs identified from the ER data.**
(EPS)

Figure S15 **ER motif identified by rGADEM and visualized with MotIV from the FOXA1 data.** The motif matches and associated E-values are based on the JASPAR database included in MotIV.
(EPS)

Figure S16 **Venn diagram for the number of overlapped occurrences of FOXA1 primary motifs.**
(EPS)

Figure S17 **Venn diagram for the number of overlapped occurrences of ER primary motifs.**
(EPS)

Figure S18 **Venn diagram for the number of overlapped occurrences of CTCF primary motifs.**
(EPS)

Figure S19 **Venn diagram for the number of overlapped occurrences of STAT1 primary motifs.**
(EPS)

Figure S20 **Example of a MotIV alignment output based on the FOXA1 data.**
(EPS)

Table S1 **GO Analysis for the ER data.**
(PDF)

Table S2 **GO Analysis for the FOXA1 data.**
(PDF)

Table S3 **GO Analysis for the CTCF data.**
(PDF)

Table S4 **GO Analysis for the STAT1 data.**
(PDF)
