# Supplementary figures and images for: An Integrated Pipeline for the Genome-Wide Analysis of Transcription Factor Binding Sites from ChIP-Seq
Source: PLoS One. 2011 Feb 16;6(2):e16432. doi: 10.1371/journal.pone.0016432 (PMC3040171; doi:10.1371/journal.pone.0016432)

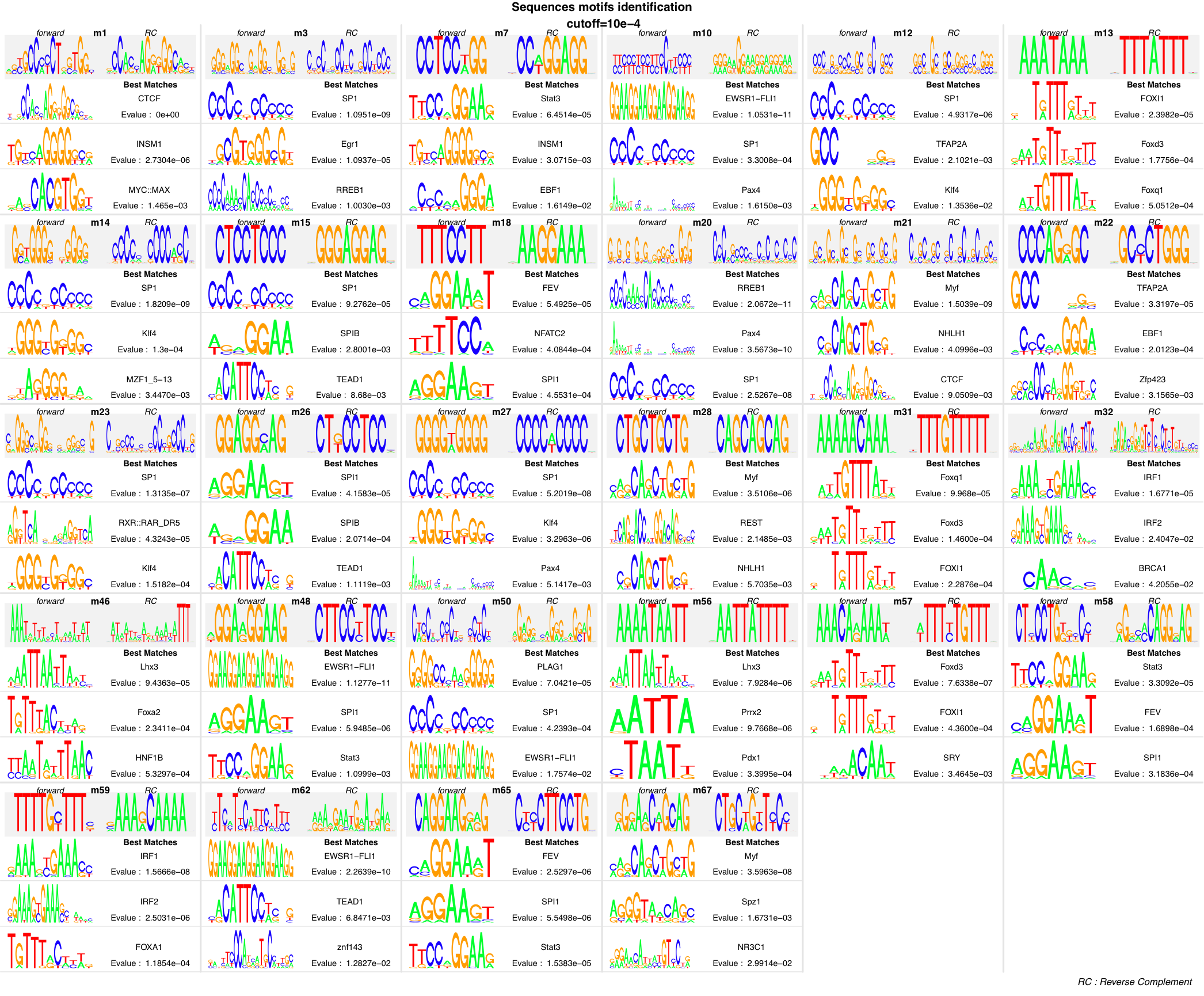

Supplement: Figure S4 — Motifs identified by rGADEM and visualized with MotIV from the CTCF data. The motif matches and associated E-values are based on the JASPAR database included in MotIV. For clarity only motifs with E-value less than 10−4 are retained. (TIFF) [file pone.0016432.s004.tiff]

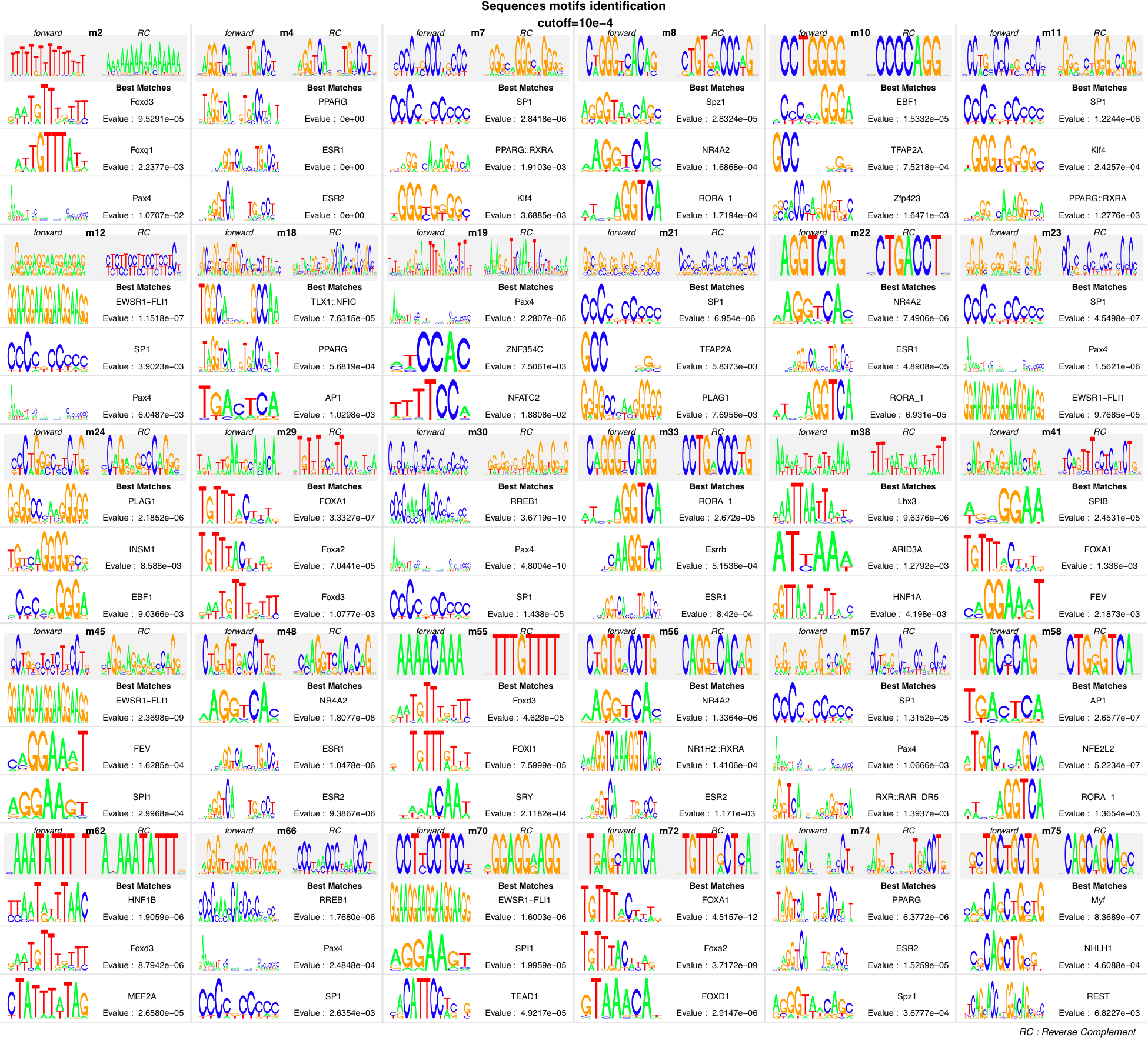

Supplement: Figure S5 — Motifs identified by rGADEM and visualized with MotIV from the ER data. The motif matches and associated E-values are based on the JASPAR database included in MotIV. For clarity only motifs with E-value less than 10−4 are retained. (TIFF) [file pone.0016432.s005.tiff]
