# Supplementary material for: An Integrated Pipeline for the Genome-Wide Analysis of Transcription Factor Binding Sites from ChIP-Seq
Source: PLoS One. 2011 Feb 16;6(2):e16432. doi: 10.1371/journal.pone.0016432 (PMC3040171; doi:10.1371/journal.pone.0016432)
Supplement: Table S1 — GO Analysis for the ER data. (PDF) [file pone.0016432.s021.pdf]

	ER	ER – AP1	ER – FOXA1
Biological process			• cell death<br>• regulation of muscle tissue development<br>• regulation of retinoic acid receptor signaling pathway
Molecular function	• transcription factor activity<br>• muscle cell development and activityt<br>• regulation of DNA replication	• growth factor activity	• transcription factor activity<br>• estrogen receptor activity
Cellular component	• contractile fiber, myofibril, actin cytoskeleton<br>• axon, cell projection part	• cytoplasm, mitochondrion<br>• cell projection membrane	• neuron projection terminus<br>• vesicle, Golgi membrane
