# Supplementary material for: An Integrated Pipeline for the Genome-Wide Analysis of Transcription Factor Binding Sites from ChIP-Seq
Source: PLoS One. 2011 Feb 16;6(2):e16432. doi: 10.1371/journal.pone.0016432 (PMC3040171; doi:10.1371/journal.pone.0016432)
Supplement: Table S2 — GO Analysis for the FOXA1 data. (PDF) [file pone.0016432.s022.pdf]

	FOXA1	FOXA1 – AP1
Biological process	• regulation of cell migration and cellular component movement	• regulation of transcription from RNA polymerase II promoter
Molecular function		• transcription factor activity<br>• sequence-specific DNA binding
