# Supplementary material for: An Integrated Pipeline for the Genome-Wide Analysis of Transcription Factor Binding Sites from ChIP-Seq
Source: PLoS One. 2011 Feb 16;6(2):e16432. doi: 10.1371/journal.pone.0016432 (PMC3040171; doi:10.1371/journal.pone.0016432)
Supplement: Table S3 — GO Analysis for the CTCF data. (PDF) [file pone.0016432.s023.pdf]

	CTCF	CTCF – Myf
Biological process	• regulation of apoptosis<br>• regulation of cell cummunication	• blastocyst development<br>• regulation of cyclase activity
Molecular function		• growth hormone-releasing hormone receptor activity<br>• protein complex binding
