# Supplementary material for: An Integrated Pipeline for the Genome-Wide Analysis of Transcription Factor Binding Sites from ChIP-Seq
Source: PLoS One. 2011 Feb 16;6(2):e16432. doi: 10.1371/journal.pone.0016432 (PMC3040171; doi:10.1371/journal.pone.0016432)
Supplement: Table S4 — GO Analysis for the STAT1 data. (PDF) [file pone.0016432.s024.pdf]

	STAT1	STAT1 – AP1	STAT1 – CTCF
Biological process	• blood vessel and vasculature development<br>• regulation of T cell proliferation, T cell tolerance induction		• blood vessel development, vasculature development
Cellular component	• cell-substrate junction, adherens junction, cell leading edge	• plasma membrane part, catenin complex	• membrane fraction
